# Transient overexpression of TGFBR3 induces apoptosis in human nasopharyngeal carcinoma CNE-2Z cells

**DOI:** 10.1042/BSR20120047

**Published:** 2013-03-13

**Authors:** Fangfang Zheng, Kaiwen He, Xin Li, Dan Zhao, Fei Sun, Yu Zhang, Dan Nie, Xingda Li, Wenfeng Chu, Yan Sun, Yanjie Lu

**Affiliations:** *Department of Pharmacology (the State-Province Key Laboratories of Biomedicine-Pharmaceutics of China, Key Laboratory of Cardiovascular Research, Ministry of Education), Harbin Medical University, Harbin, Heilongjiang 150081, People's Republic of China; †Department of Surgery, the 2nd Affiliated Hospital, Harbin Medical University, Harbin, Heilongjiang 150081, People's Republic of China

**Keywords:** apoptosis, CNE-2Z, nasopharyngeal carcinoma (NPC), transforming growth factor type III receptor (TGFBR3), AIF, apoptosis-inducing factor, AO/EB, acridine orange/ethidium bromide, [Ca^2+^]_i_, intracellular Ca^2+^ concentration_;_ DAB, diaminobenzidine, GAPDH, glyceraldehyde-3-phosphate dehydrogenase, MTT, 3-(4,5-dimethylthiazol-2-yl)-2,5-diphenyl-2*H*-tetrazolium bromide, NC, negative control, NPC, nasopharyngeal carcinoma, PBST, PBS containing 0.1% Tween 20, PFA, paraformaldehyde, pNA, *p*-nitroanilide, TGFBR3, transforming growth factor type III receptor, TRPM, transient receptor potential melastatin, Xiap, X-linked inhibitor of apoptosis

## Abstract

NPC (nasopharyngeal carcinoma) is a common malignancy in southern China without defined aetiology. Recent studies have shown that TGFBR3 (transforming growth factor type III receptor, also known as betaglycan), exhibits anticancer activities. This study was to investigate the effects of TGFBR3 on NPC growth and the mechanisms for its actions. Effects of TGFBR3 overexpression on cell viability and apoptosis were measured by MTT [3-(4,5-dimethylthiazol-2-yl)-2,5-diphenyl-2*H*-tetrazolium bromide], AO/EB (acridine orange/ethidium bromide) staining and electron microscopy in human NPC CNE-2Z cells. The expression of apoptosis-related proteins, p-Bad, Bad, XIAP (X-linked inhibitor of apoptosis), AIF (apoptosis-inducing factor), Bax and Bcl-2, was determined by Western blot or immunofluorescence analysis. Caspase 3 activity was measured by caspase 3 activity kit and [Ca^2+^]_i_ (intracellular Ca^2+^ concentration) was detected by confocal microscopy. Transfection of TGFBR3 containing plasmid DNA at concentrations of 0.5 and 1 μg/ml reduced viability and induced apoptosis in CNE-2Z in concentration- and time-dependent manners. Forced expression of TGFBR3 up-regulated pro-apoptotic Bad and Bax protein, and down-regulated anti-apoptotic p-Bad, Bcl-2 and XIAP protein. Furthermore, transient overexpression of TGFBR3 also enhanced caspase 3 activity, increased [Ca^2+^]_i_ and facilitated AIF redistribution from the mitochondria to the nucleus in CNE-2Z cells, which is independent of the caspase 3 pathway. These events were associated with TGFBR3-regulated multiple targets involved in CNE-2Z proliferation. Therefore transient overexpression of TGFBR3 may be a novel strategy for NPC prevention and therapy.

## INTRODUCTION

NPC (nasopharyngeal carcinoma) is a relatively uncommon malignant head and neck cancer worldwide, but is highly prevalent in South China and Southeast Asia [1]. The worldwide incidence of NPC exceeds 80000 cases per year [2]. Its frequency is very high in the Guangzhou area, China, where the annual incidence reaches 25 cases per 100000 [3]. Radiotherapy can effectively control early stage NPC, yielding an excellent 90–95% 5-year local control rate in clinical trials. However, radiotherapy alone is not the optimal treatment for patients with locally advanced disease, for it yields an unsatisfactory 5-year survival rate of only approximately 50% [4]. For this reason, platinum-based chemotherapy combined with 5-FU (5-fluorouracil) has been the most common strategy for NPC, but its efficacy is no more than 50–60% [5].

TGF-β superfamily ligands function as tumour suppressors through their ability to inhibit cell proliferation, maintain tissue architecture, inhibit genomic instability and induce senescence and apoptosis [6]. Recently, TGFBR3 (transforming growth factor type III receptor, also known as betaglycan) has been established as a suppressor of cancer progression. A growing body of studies have shown that TGFBR3 is lost in a variety of cancers, including human kidney [7], pancreas [8], prostate [9], lung [10], breast [11] and ovary carcinomas [12]. Loss of expression of TGFBR3 correlates with disease progression and a poor prognosis, and restoration of its expression in cancer cells exerts a regulatory role in cell migration, invasion, angiogenesis and metastasis [13]. However, the physiological and pathophysiological roles of TGFBR3 in NPC cells and the signalling mechanisms remained yet to be determined.

In this study, we demonstrated that TGFBR3 is expressed in CNE-2Z NPC cells. Transient transfection of TGFBR3 significantly decreased viability and induced apoptosis in these cells, with significant activation of caspase 3-dependent and -independent signalling pathways and elevation of [Ca^2+^]_i_ (intracellular Ca^2+^ concentration) as well.

## MATERIALS AND METHODS

### Reagents

pc-DNA3.1-mTGFBR3 plasmid was purchased from GeneChem Co., Ltd. Primary antibodies for Bax, Bcl-2, Bad, p-Bad, AIF (apoptosis-inducing factor), Xiap (X-linked inhibitor of apoptosis) protein and TGFBR3 were from Cell Signaling. Other chemicals were from Sigma.

### CNE-2Z cell culture and TGFBR3 transfection

CNE-2Z, a human NPC cell line, was provided by Harbin Medical University and cultured in RPMI 1640 medium containing 10% (v/v) heat-inactivated FBS (fetal bovine serum) at 37°C in an incubator containing humidified air with 5% (v/v) CO_2_. Cells were transfected with pc-DNA3.1-mTGFBR3 plasmid at a dosage of 0.5 or 1 μg/ml for 48 h using Lipofectamine™ 2000.

### Cell viability assay

Cells were seeded on to 96-well plates at 1×10^4^ cells per well 24 h before treatment. The cells were transfected with TGFBR3 (0.5 or 1 μg/ml). After 48 h, 15 μl (5 mg/ml) MTT [3-(4,5-dimethylthiazol-2-yl)-2,5-diphenyl-2*H*-tetrazolium bromide] (Sigma-Aldrich) was added to each well, and incubated at 37°C for 4 h. Then the MTT solution was removed and 150 μl of DMSO was added to dissolve the crystals. The mixtures were shaken for 10 min to fully dissolve the crystals. A microplate reader (TECAN) was used to measure the absorbance at a wavelength of 490 nm. Cell viability was expressed as percentage changes of the absorbance values from the treatment group over the control group.

### Electron microscopy

CNE-2Z cells were cultured in 60 mm plates, collected in PBS solution and fixed with 2% (v/v) PFA (paraformaldehyde) containing 2.5% (w/v) glutaraldehyde (Paesel-Lorei) buffered in HMSS (Hank's modified salt solution) at 4°C for 4 h. The cells were further fixed in 1% (w/v) OSO_4_ solution buffered by 0.1 M cacodylate (pH 7.2) at 4°C for 2 h, and then the cells were scraped-off from the plastic and dehydrated in ethanol. Dehydration was completed in propylene oxide. The specimens were embedded in Araldite (Serva). Ultrathin sections were produced on an FCR Reichert Ultracut ultramicrotome (Leica), and mounted on pioloform-coated copper grids and contrasted with lead citrate. Specimens were analysed and documented with an EM 10A electron microscope (Zeiss).

### AO/EB (acridine orange/ethidium bromide) fluorescence staining

CNE-2Z cells in the exponential growth phase were cultivated on sterile coverslips for 24 h, and were subsequently transfected with 0.5 or 1 μg/ml TGFBR3 for 48 h. The cells were washed twice with PBS, and then mixed with 1 ml of dye mixture containing 100 mg/ml AO and 100 mg/ml EB in PBS [14]. Cellular morphological changes were examined by using fluorescence microscopy (×100). The percentage of apoptotic cells was calculated by the following formula: apoptotic rate (%)=number of apoptotic cells/number of all cells counted [15,16].

### Western blotting analysis

Total protein samples were extracted from CNE-2Z cells by standard lysis buffer: 50 mM Tris (pH 7.4), 150 mM NaCl, 1% (v/v) Triton X-100, 1% (w/v) sodium deoxycholate, 0.1% (w/v) SDS and sodium orthovanadate, sodium fluoride, EDTA and leupeptin and protease inhibitor at the ratio of 1:100. Samples were quantified by the BCA (bicinchoninic acid) Protein Assay Reagent (Pierce) method, and 80 μg of protein was separated by SDS/10–12% PAGE loading buffer with 5% (v/v) 2-mercaptoethanol, and loaded in 10–12% PAGE gels. The proteins were then transferred to nitrocellulose membranes. After blocking with 5% (w/v) non-fat dried skimmed milk powder in PBST [PBS containing 0.1% (v/v) Tween 20], the membranes were incubated with primary antibodies for TGFBR3 (1:500 dilution), Bax (1:500 dilution), Bcl-2 (1:500 dilution), Bad (1:500 dilution), p-Bad (1:500 dilution), XIAP (1:500 dilution) and GAPDH (glyceraldehyde-3-phosphate dehydrogenase; 1:1000 dilution) at 37°C for 2 h. Then the membranes were washed twice with PBST and incubated with a secondary antibody conjugated with polymeric HRP (horseradish peroxidase; Wuhan Boster) for 1 h at room temperature (21–23°C). The membranes were washed again with PBST four times, and immune detection was performed by adding 10 ml of 0.25% (w/v) DAB (diaminobenzidine) in 0.01 citric acid buffer solution, plus 10 μl of 30% (v/v) H_2_O_2_. The DAB solution was prepared immediately before use. The obtained digital images of Western blotting results were then used for densitometry measurements (Gel-Pro analyser 4.0, Media Cybernetics). Western blotting bands were quantified using Odyssey v3.0 software by measuring the band intensity (area×absorbance) for each group and normalizing to GAPDH band as an internal control.

### Immunofluorescence staining

#### Localization of AIF

To analyse cellular localization of AIF, cells were cultured on coverslips and treated with rabbit anti-AIF antibody (1:100 dilution) overnight at 4°C. Then the cells were fixed with a freshly prepared PFA solution (4% in PBS, pH 7.4) for 10 min at room temperature, and permeabilized with 0.01% (v/v) Triton X-100 for 10 min on ice. The secondary antibodies used were conjugated with Alexa Fluor® 594 (Mobitech) or FITC 488 (Invitrogen), and incubated for 1 h at room temperature. DAPI (4′,6-diamidino-2-phenylindole; Calbiochem) was further used for nucleus staining. The cells were examined with a fluorescence microscope.

#### Caspase 3 activity assay

Caspase 3 activity was analysed using a caspase 3 activity assay kit (Beyotime, China) according to the manufacturer's instructions, using substrate peptides Ac-DEVD-pNA (*p*-nitroanilide), Ac-IETD-pNA and Ac-LEHD-pNA, respectively. Briefly, the supernatant of cell lysate was mixed with buffer containing the substrate peptides for caspase attached to pNA. The release of pNA was quantified by determining the absorbance with an ELISA reader at 405 nm. The caspase activities were expressed as percentage over control.

#### Intracellular Ca^2+^ measurement

Cytosolic free Ca^2+^ was measured by confocal microscopy [17]. The CNE-2Z cells adherent to the coverslips were transfected with 1 μg/ml TGFBR3 in the presence of 5 μM 2-APB for 48 h. The cells were then washed once with the standard Tyrode solution (in mM: 126 NaCl, 5.4 KCl, 10 Hepes, 0.33 NaH_2_PO_4_·2H_2_O, 1.0 MgCl_2_·6H_2_O, 1.8 CaCl_2_ and 10 glucose, pH adjusted to 7.40), and incubated in the working solution containing 20 mM Fluo-3/AM (Molecular Probes) and 0.03% (w/v) Pluronic F-127 at 37°C for 40 min. The cells were washed once with the standard Tyrode solution. The images were captured by a confocal microscope (488 nm for λ_ex_, 530 nm for λ_em_). Forty-eight hours after the TGFBR3 transfection, fluorescent intensity was measured.

### Data analysis

Data are shown as means±S.D. of three to six independent experiments and were evaluated by unpaired Student's *t*-test. Differences were considered to be significant at *P*<0.05.

## RESULTS

### Overexpression of TGFBR3 suppresses the viability of CNE-2Z cells

**Figure 1:**
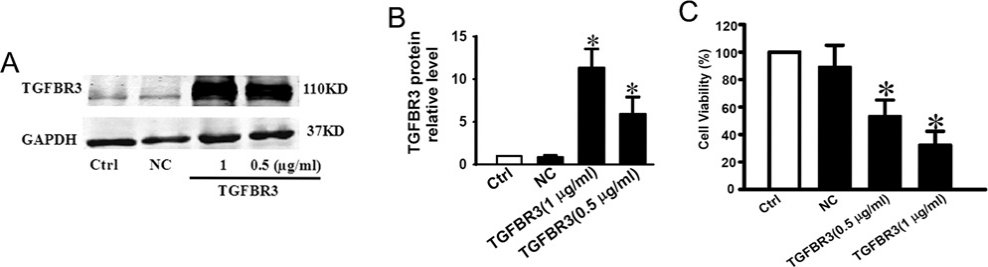
Overexpression of TGFBR3 inhibits survival of CNE-2Z cells CNE-2Z cells were transfected with TGFBR3 DNA at concentrations of 0.5 μg/ml and 1 μg/ml. NC represents empty vectors (1 μg/ml of pc-DNA3.1 plasmid) transfected into CNE-2Z cells which serves as a NC. (**A**) TGFBR3 expression determined by Western blot analysis. (**B**) Averaged band density from three independent experiments. (**C**) Relative cell viability determined by MTT assay. *P<0.05 compared with Ctrl, *n*=6 independent experiments for each condition.

Successful transfection of TGFBR3 was verified by our data shown in Figures 1(A) and 1(B). Western blotting showed that TGFBR3 protein level was increased in a concentration-dependent manner in cells treated with 0.5 μg/ml (7-fold) and 1 μg/ml (11.5-fold) of TGFBR3 plasmid DNA (Figures 1A and 1B). Figure 1(C) shows that the viability of CNE-2Z cells transfected with 0.5 or 1 μg/ml TGFBR3 DNA was reduced by 53.1±7.9 and 46.9±7.0%, respectively.

### Overexpression of TGFBR3 induces apoptosis in CNE-2Z cells

**Figure 2:**
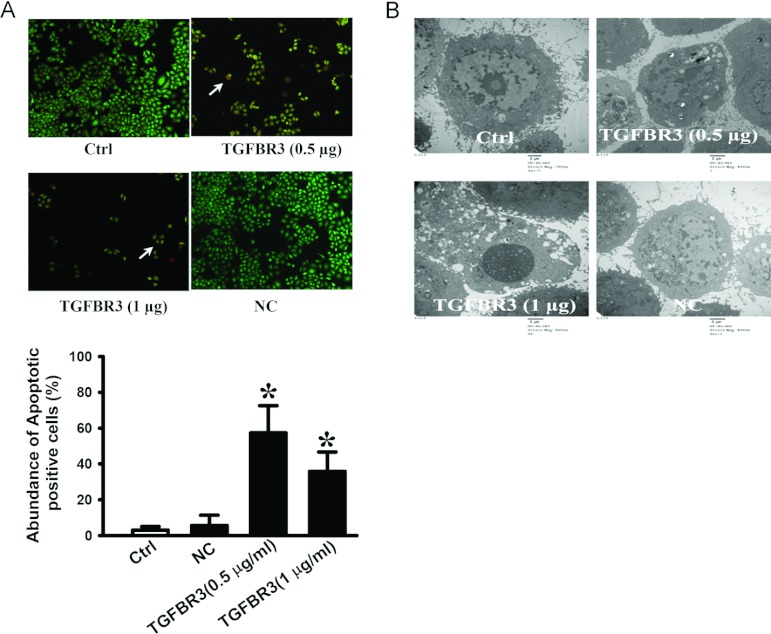
Overexpression of TGFBR3 induces apoptosis in CNE-2Z cells (**A**) AO/EB staining to detect changes in nucleus. White arrows indicate apoptotic cells. (**B**) Transmission electron microscopy to estimate micro-morphological changes (magnification: ×8000). Statistical bar graph of apoptotic cells by AO/EB staining. Arrows indicate apoptotic cells. *P<0.05 compared with Ctrl. *n* = 3 independent experiments for each group.

Cell viability is a balance of proliferation and apoptosis [18]. To investigate whether TGFBR3 regulates apoptosis, AO/EB staining and electron microscopy were used to detect the apoptotic cells. The results from our fluorescence microscopic analysis are shown in Figure 2(A). Three types of cells were recognized under a fluorescence microscope: live cells (green), apoptotic cells (yellow) and necrotic cells (red). Forced expression TGFBR3 induced substantial apoptotic cells (*P*<0.05), and transfection of the empty vector failed to do so (*P*>0.05). Under an electron microscope the cells with TGFBR3 overexpression exhibited robust changes in micro-structure, including cell surface microvilli reduction, nuclear chromatin condensation, margination and membrane blistering (Figure 2B).

### Overexpression of TGFBR3 activates proapoptotic signalling pathways

**Figure 3:**
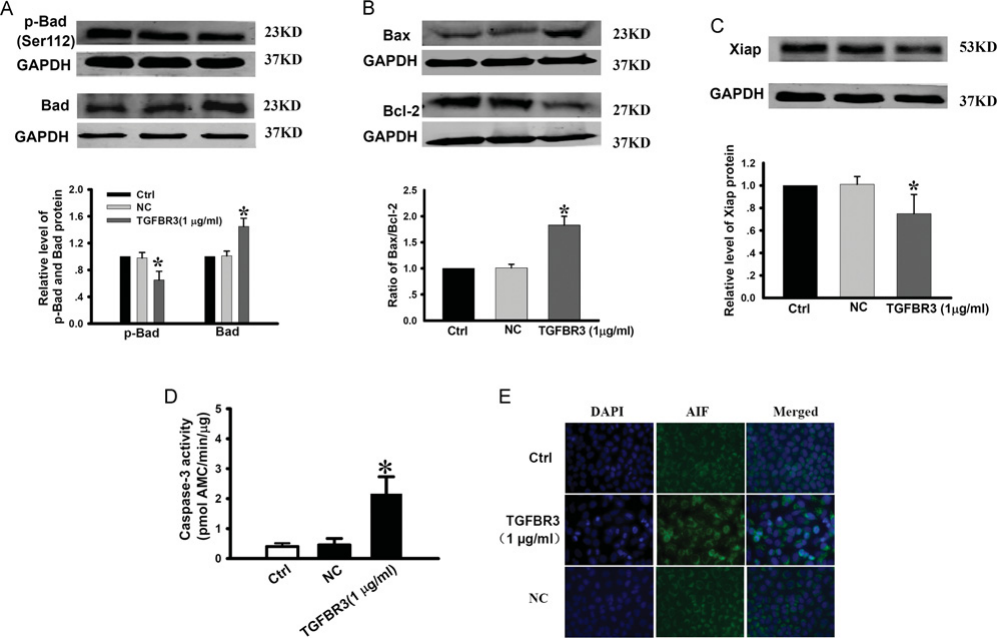
Overexpression of TGFBR3 alters p-Bad, Bad, Bax, Bcl-2 and XIAP expression, and promotes caspase 3 activation and AIF release from mitochondria to nucleus Western blotting was used to detect p-Bad, Bad, (**A**), Bax, Bcl-2 (**B**) and XIAP (**C**) expression in CNE-2Z cells transfected with TGFBR3. Relative expression of Bax, Bcl-2, p-Bad, Bad and XIAP was normalized to GAPDH. *n*=3 independent experiments for each group. (**D**) Activation of caspase 3 by TGFBR3 overexpression. Data are averaged from five independent experiments for each group. (**E**) Immunofluorescence images of AIF by confocal microscopy (original magnification, ×400). Similar results were observed from another three experiments. *P<0.05 compared with Ctrl.

To explore the mechanisms by which TGFBR3 overexpression induces apoptosis in CNE-2Z cells, we measured the downstream proteins of the TGFBR3 apoptotic pathway, including Bax, Bcl-2 Bad, p-Bad and XIAP. Figure 3 demonstrates that forced expression of TGFBR3 up-regulated Bad and Bax, and down-regulated p-Bad and Bcl-2 expression (Figures 3A and 3B). The anti-apoptotic protein XIAP was also detected in CNE-2Z cells, and overexpression of TGFBR3 decreased XIAP protein expression (Figure 3C). Empty vector did not affect Bax, Bcl-2, Bad, p-Bad and XIAP protein levels. In addition, relative caspase 3 activity was significantly increased 2.2-fold by TGFBR3 transfection, but no obvious change was seen with the NC (negative control) (Figure 3D). To determine potential toxic effects of TGFBR3, the cellular distributions of AIF were detected by immunofluorescence staining. As shown in Figure 3(E), the Alexa Fluor® signals indicated that overexpression of TGFBR3 facilitated AIF redistribution from the mitochondria to the nucleus, which is independent of the caspase 3 pathway.

### Overexpression of TGFBR3 increases [Ca^2+^]_i_

**Figure 4:**
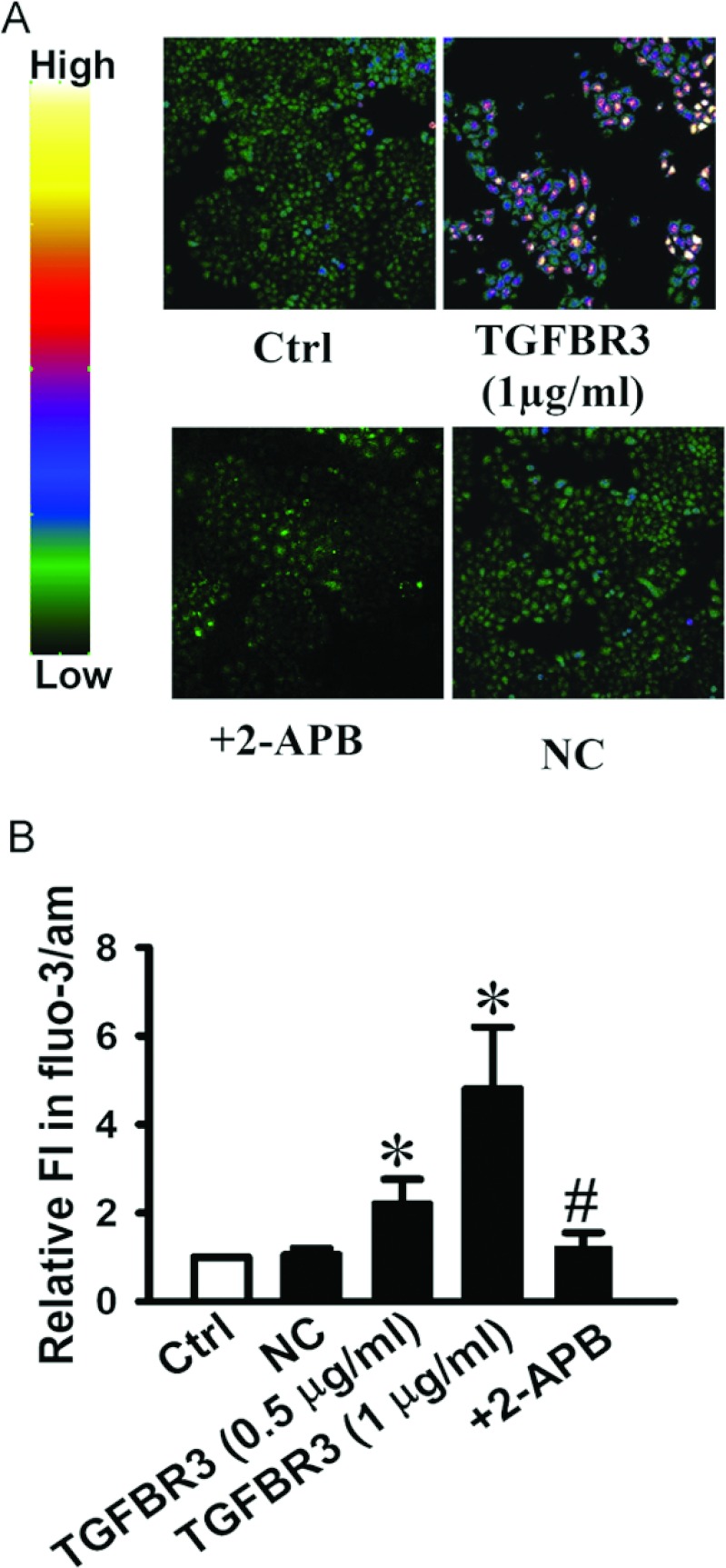
Overexpression of TGFBR3 induces elevation of [Ca^2+^]_i_ in CNE-2Z cells (**A**) Representative confocal images of [Ca^2+^]_i_ in CNE-2Z cells (magnification: ×200). (**B**) Summarized results of fluorescence intensity obtained from CNE-2Z cells under the indicated condition. Data are averaged from six experiments for each condition (*n*=6). *P<0.05 compared with Ctrl; # *P*<0.05 compared with TGFBR3.

Increase of [Ca^2+^]_i_ is known to be a trigger for apoptosis [19]. Confocal microscopy was used to measure the changes of [Ca^2+^]_i_. Figure 4(A) shows that TGFBR3 overexpression triggered the elevation of [Ca^2+^]_i_ in a concentration-dependent manner. [Ca^2+^]_i_ was increased 4.6-fold in CNE-2Z cells transfected with 1 μg/ml TGFBR3 for 48 h, compared with the control (*P*<0.05). Moreover, pretreatment with 5 μM 2-APB, a blocker of TRPM7 (transient receptor potential melastatin 7) channel, prevented the elevation of [Ca^2+^]_i_ (Figure 4B).

## DISCUSSION

Our present incomplete understanding of the precise mechanisms for the dual tumour suppressor/tumour promoter roles of the TGF-β superfamily ligands represents a major obstacle to targeting these molecules for the treatment of human cancer [20]. In this study, we identified a novel cellular function of TGFBR3: the potent anti-proliferative effects on CNE-2Z cells by causing apoptosis. We further elucidated activation of both caspase 3-dependent and -independent pathways and intracellular Ca^2+^ overload as important mechanisms for TGFBR3-induced apoptosis and thereby suppression of NPC cancer cell growth. Therefore the current study may provide a new insight into the pathophysiological role of TGFBR3 in NPC diseases.

Previous studies have shown that TGFBR3 produces anticancer activities via regulating invasion, proliferation, cell migration and angiogenesis and that expression of TGFBR3 is decreased in a variety of malignancies such as breast, kidney, lung, ovary, pancreas and prostate cancers [20]. On the other hand, another study from Gatza et al. [20] in 2010 also demonstrated that TGFBR3 expression is not significantly altered at the mRNA level but is increased at the protein level, and this increase promotes tumorigenicity due to TGFBR3-mediated resistance to ligand and chemotherapy-induced apoptosis, increased anchorage-independent cell growth, and increased cell migration. Similarly, our previous study also showed that overexpression of TGFBR3 protects cardiac fibroblasts from hypoxia-induced apoptosis. It seems that whether TGFBR3 promotes or inhibits cell proliferation may depend upon the cell type; different cell types have different predominant signalling pathways to apoptosis. Several studies have reported that Bax, Bcl-2 and caspase 3 are the key molecules participating in apoptosis in NPC cells. For instance, celecoxib combined with radiotherapy up-regulates Bax and caspase 3 protein expression and down-regulates Bcl-2 expression in CNE-2 cells [21]. *Selaginella doederleinii* extract triggers apoptosis by increasing the ratio of Bax/Bcl-2 in CNE cells [22]. Curcumin-induced apoptosis was accompanied with up-regulation of Bax and down-regulation of Bcl-2, and consequent dysfunction of mitochondria leading to activation of caspase 3 in NPC-TW 076 cells [23]. Astragalus increased caspase 3 and Bax protein levels, and decreased the Bcl-2 protein level in NPC cell line CNE-2Z [24]. Resveratrol induces NPC cell apoptosis by activating caspase 3 via up-regulating Bax and down-regulating Bcl-2 [25]. These results are consistent with our finding that TGFBR3 activates caspase 3 to induce apoptosis by regulating Bcl-2 and Bax expression in NPC cells. It is known that Bad, a member of the Bcl-2 family, normally binds to the Bcl-2/Bcl-X complex and triggers apoptosis. However, phosphorylated Bad will dissociate from this complex, resulting in release of Bcl-2/Bcl-X and suppression of apoptosis [26]. XIAP interacts with caspase 3 to block its full activation, substrate cleavage and cell death [27]. To fully understand how TGFBR3 increases the ratio of Bax over Bcl-2 and activation of caspase 3, we detected the level of Bad, p-Bad and XIAP protein. Here, we found TGFBR3 treatment significantly increased levels of Bad and decreased levels of p-Bad and XIAP. These data indicated that caspase 3-dependent apoptotic signalling plays a critical role in the anticancer activity of TGFBR3.

Although caspases have been recognized as important mediators of apoptosis, there is accumulating evidence for the existence of caspase-independent mechanisms of cell death. AIF is a putative caspase-independent effector of cell death that has recently been cloned and characterized. AIF is a mitochondrial intermembrane flavoprotein that has been reported to be released from the mitochondria and to be translocated to the nucleus in response to specific death signals. We observed that TGFBR3 resulted in AIF release from mitochondria to nucleus, suggesting that the caspase 3-independent signalling pathway is involved in the apoptosis induced by TGFBR3. Moreover, one of the biochemical events in cell differentiation, proliferation, growth and apoptosis is elevation of [Ca^2+^]_i_. Radiation-resistant capacity of NPC was shown to be mostly due to improved cellular Ca^2+^ homoeostasis promoting anti-apoptosis and DNA repair and rescuing tumour cells from cell death under radiation therapy [28]. Tanshinone II was reported to exert its anticancer effect by increasing [Ca^2+^]_i_ concentration to induce apoptosis in CNE cells [29]. Rhein induces apoptosis in NPC cells through Ca^2+^-dependent mitochondrial death pathway [30]. In agreement with these studies, we found here that overexpression of TGFBR3 elevated [Ca^2+^]_i_ in CNE-2Z cells. Some studies showed that TRPM7, a Ca^2+^-permeable non-selecting cation channel, plays an important role in migration of NPC cells [31,32]. We also found that 2-APB, which is a blocker for the TRPM7 channel, prevented Ca^2+^ overload induced by TGFBR3, suggesting that TRPM7 is possibly involved in the increase of [Ca^2+^]_i_ induced by TGFBR3. However, the precise mechanism for TGFBR3 to cause Ca^2+^ overload in CNE-2Z cells is still unknown, which is of considerable interest for us to investigate in our future study.

In conclusion, our study demonstrated for the first time that overexpression of TGFBR3 markedly inhibits cell proliferation by inducing apoptosis in CNE cells, This event is likely to be associated with TGFBR3-regulated multiple targets in CNE-2Z cell proliferation. TGFBR3 increased the expression of Bad and decreased the level of p-Bad and subsequently mediated increase of the ratio of Bax over Bcl-2, leading to activation of caspase 3. Our findings suggest that TGFBR3 overexpression is a potential approach for clinical treatment of NPC, provided that TGFBR3 gene therapy could be applied to the right types of NPC with the right cellular context to produce the right consequence with minimal ‘off-target’ effects.
